# Stress concentration-relocating interposer in electronic textile packaging using thermoplastic elastic polyurethane film with via holes for bearing textile stretch

**DOI:** 10.1038/s41598-022-13493-7

**Published:** 2022-06-03

**Authors:** Seiichi Takamatsu, Suguru Sato, Toshihiro Itoh

**Affiliations:** 1 Graduate School of Engineering, The University of Tokyo, 7-3-1 Hongo, Bunkyo-ku, Tokyo, 113-8656 Japan; 2 Graduate School of Frontier Sciences, The University of Tokyo, 5-1-5 Kashiwanoha, Kashiwa-shi, Chiba-ken 277-8561 Japan

**Keywords:** Electrical and electronic engineering, Mechanical engineering

## Abstract

Electronic textile (e-textile) devices require mechanically reliable packaging that can bear up to 30% stretch induced by textile crimp stretch, because the boundary between the rigid electronic components and the soft fabric circuit in the e-textile is prone to rupture due to mismatch of their mechanical properties. Here, we describe a thin stress-concentration-relocating interposer that can sustain a textile stretch of up to 36%, which is greater than the 16% stretch of conventional packaging. The stress-concentration-relocating interposer consists of thin soft thermoplastic polyurethane film with soft via holes and is inserted between the electronic components and fabric circuit in order to move the area of stress concentration from the wiring area of the fabric circuit to the film. A finite element method (FEM) simulation showed that when the fabric is stretched by 30%, the boundary between the electrical components and the insulation layer is subjected to 90% strain and 2.5 MPa stress, whereas, at 30% strain, the boundary between the devices and the wiring is subjected to only 1.5 MPa stress, indicating that the concentration of stress in the wiring is reduced. Furthermore, it is shown that an optimal interposer structure that can bear a 30% stretch needs insulating polyurethane film in excess of 100 μm thick. Our thin soft interposer structure will enable LEDs and MEMS sensors to withstand stretching in several types of fabric.

## Introduction

E-textiles, in which sensors and electronic devices are incorporated in fabric, have potential applications in entertainment, communications^1^ robotics^2^, and virtual reality^3–6^, and healthcare^5,7–10^. In particular, e-textiles can be used to make costumes that integrate LEDs and can be the basis of digital healthcare wearables that collect various healthcare data such as electrocardiogram, myoelectricity, body temperature, and oxygen saturation level. Other applications include smart wear with virtual reality controllers, haptic interface devices, and other functions ^11^. However, when highly functional sensors and actuators, such as vibration motors, are implemented in textiles, they must be able to withstand not only bending but also stretching when the textile crimps, as the people wearing the smart wear will move or even play sports or dance. The resultant crimp stretch of less of than 5% is derived from the plain weaving structure of the textile. In case of knitwear, the e-textile should withstand stretching of more than 30%. Previous studies have attempted to incorporate stretchable electronic devices in electronic textiles, for example, island-bridge devices in which the island consists of rigid electronic components, such as LSI, MEMS sensors, resistors, or other passive components, and stretchable bridge wiring is made from stretchable silver paste or wavy metal interconnects. Stretchable silver paste, which is a mixture of silver and rubber, has been used as a stretchable interconnection in electronic textiles^12–14^. Meanwhile, there are few stretchable electronic components, because it is difficult to maintain the properties of electronic functional materials such as semiconductors by blending them with rubber.

Island-bridge structure devices with rigid electronic components and stretchable silver paste have been utilized in applications including LED lighting, ultrasonic probes, and Internet of Things edge devices. However, when an island bridge structure is stretched, a serious problem arises; the interconnection at the boundary between the island and the bridge of the wiring is prone to breakage^5,15–18^. The modulus of elasticity of cloth and soft stretchable wiring is a few MPa, while the modulus of elasticity of plastic substrates and electronic components is several GPa to several hundred GPa. Therefore, strain concentrates at the boundary between the fabric substrate and the wiring and chip, where the modulus of elasticity varies by more than 1000 times, and the stretchable silver paste ruptures at the boundary under tension.

Previous research aimed at preventing stress from concentrating at the boundaries of the rigid electric components and the stretchable silver paste in textiles has focused on trying to vary Young's modulus smoothly between the rigid electronic device and the elastic interconnection^18^.One approach is to change Young's modulus by changing the thickness of the protective rubber layer^19,20^. Here, thick polydimethylsiloxane (PDMS) or some other encapsulation material (about 5 mm thick) is attached to the top of the electronic component, while the PDMS on the stretchable silver paste or wavy metal interconnect is thinned over a certain distance to about 1 mm; i.e., the thickness of the PDMS between the rigid component and the interconnect smoothly varies ^19,21,22^. However, since the PDMS protective layer has to be several millimeters thick to change Young's modulus, the components in the fabric are uncomfortable to wear.

Another approach is to change the hardness as well as the thickness of the PDMS; this method has been used to make an organic thin film transistor^19,23^. The stiffness under the transistor chip was hardened by using UV-curable PDMS, while the region under the wiring was softened. The hardness of the remaining part was smoothly varied by changing the UV exposure intensity^24^. Although thickness of the substrate in this case was less than 1 mm, it was concluded that it would be time consuming to expose different parts of the material to different UV illuminations or durations especially when making devices that have large areas or to apply this method to mass production of stretchable circuits in textiles.

**Figure 1:**
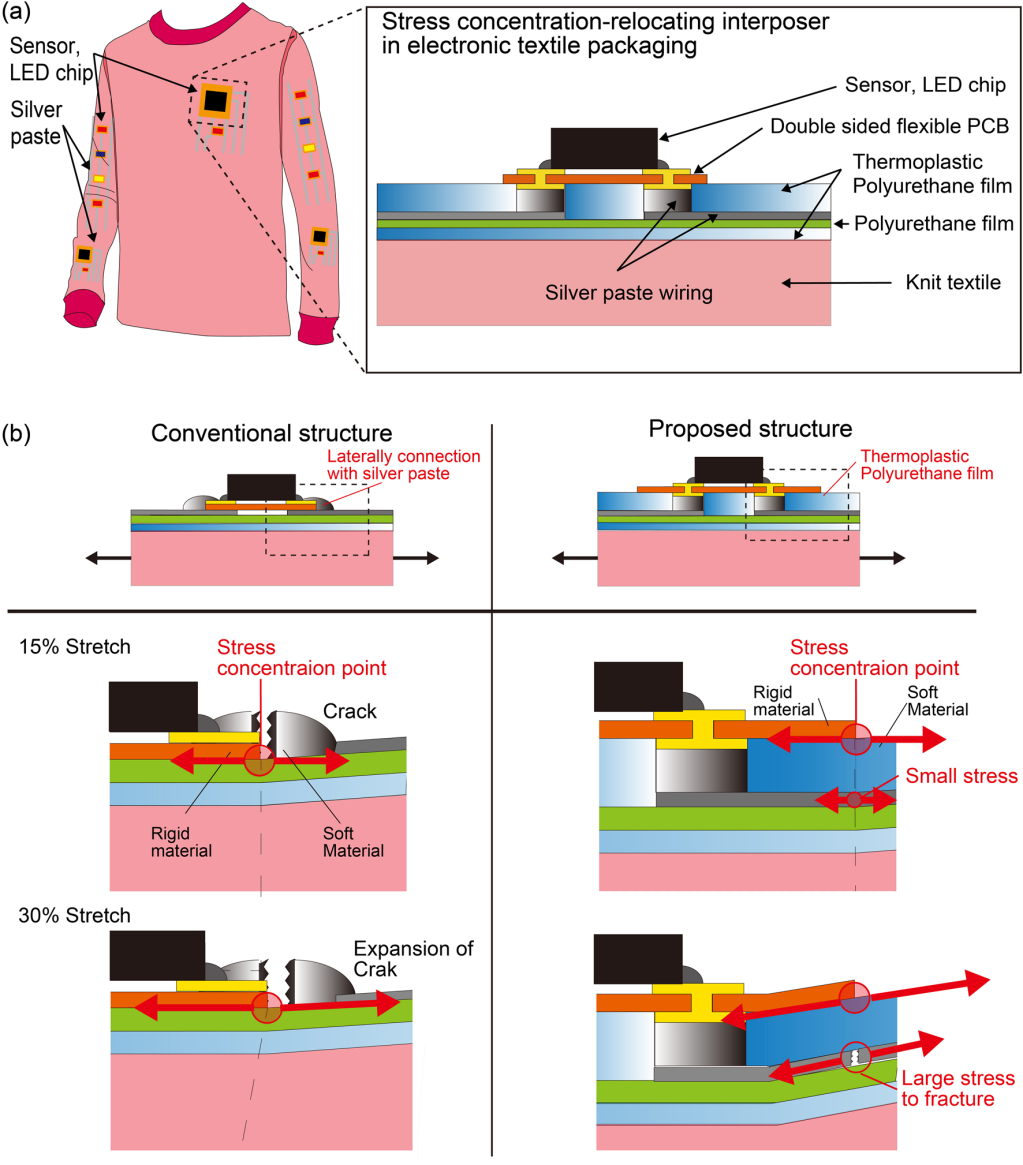
(**a**) Smartwear with the proposed stress-concentration-relocation electronic textile packaging structure. The electronic components are placed on thermoplastic layers. (**b**) The area of stress concentration is in the silver paste wiring in the conventional structure but in the thermoplastic polyurehtane interposer layer in the proposed structure.

The above discussion points to a need for a new electronic-textile device packaging that can reduce stress concentration. In particular, the packaging structure should be thin and able to be applied to a large area. Here, to solve the problem of stress concentration due to mismatches between the characteristics of rigid electronic components and soft silver paste electrodes, we propose to use an interposer structure in which the stress concentration area is moved from the stretchable silver paste wiring to the insulating layer. The interposer consists of soft thermoplastic polyurethane film perforated with via holes. Soft thermoplastic polyurethane as thin as 100 μm works as an adhesive and is inserted between the FPC mounting the electronic components and the fabric circuit with stretchable silver paste as shown in Fig. 1a. The via holes connect the electronic components and the fabric wiring. Thermoplastic polyurethane has the advantage of being able to be fabricated in very thin (100 μm) sheets and is easy to commercialize, as it is used as an adhesive for ironing appliqués on clothes. It can also be used to mount chips of different designs by drilling via holes with a laser. Furthermore, its difference from other stress-concentration-reducing structures is that instead of reducing the stress of the entire board, it relocates the stress concentration to the part of the soft thermoplastic polyurethane interposer layer that is not the wiring. The mechanical properties of this structure were evaluated in experiments and in finite element simulations. Conventional proposed structures were manufactured and evaluated in tensile tests to see if they could sustain stretching of more than 30%. Then, a prototype stretchable electronic textile device with LEDs and MEMS accelerometers was demonstrated.

The proposed structures are shown in Fig. 1a. A soft thermoplastic polyurethane film with an adhesive function was placed between a double-sided flexible substrate on which electronic components were mounted and a fabric circuit with stretchable silver paste. The fabrication of the proposed structure began with screen printing of silver paste (SSP2801, Toyobo Co., Ltd.) on polyurethane (PU) film (DUS202, SSP2801, Toyobo Co., Ltd.) by using a screen-printing machine (DP-320, Newlong Seimitsu Kogyo Co., Ltd.). The fabric circuit board was formed by using a hot press to bond the PU film on the fabric (ALPHA Wiper TX1009, TexWipe) using thermoplastic polyurethane (DUS202, Sheedom Co., Ltd.). LEDs and MEMS components were mounted on a double-sided flexible circuit board (Unicraft corp.). The proposed interposer consisted of 100-μm-thick thermoplastic polyurethane film with via holes made by laser cutting. After the interposer was placed on the fabric circuit and the via holes were filled with silver paste, the FPC mounting the electronic components was bonded on the interposer.

Figure 1b compares stress concentrations in the conventional structure and in the proposed structure when tension is applied to the electronic textile devices. In the conventional packaging structure, the electronic components, which are soldered on a single-sided FPC, are laterally connected to the silver paste wiring of the fabric circuit. The stress concentrates at the boundary between the rigid FPC and the soft silver paste wiring when tension is applied to the textile substrate. This is because the flexible printed circuit board, which is harder than the PU film and the stretchable silver paste, is not distorted by the tension, meaning that the strain is applied only to PU film and stretchable silver paste, especially the boundary of the paste and the FPC; this leads to breakage. In particular, even if the paste has high stretchability, the fabric circuit breaks under an applied strain of 15%. On the other hand, in the proposed structure, in which a soft thermoplastic polyurethane film-based interposer is inserted between the rigid FPC and soft fabric circuit with silver paste, the stress concentrates in the insulation layer of the thermoplastic polyurethane film, not in the fabric circuit. As shown in Fig. 1b, in case of a strain of 15%, the strain applied to the silver paste of fabric circuit is small. On the other hand, when the strain reaches 30%, the stress at the silver paste of the fabric circuit increases, causing the wiring to break.

## Methods

### Fabrication of stress relocation interposer of electronic textile packaging structure

The packaging structure was fabricated in three steps.

(a) The circuit pattern was fabricated by screen-printing on a 100-μm-thick polyurethane sheet (DUS202, Sheedom Co., Ltd.) with stretchable conductive paste (SSP2801, Toyobo Co., Ltd.). The screen mask was made by Mitani Micronics Co., Ltd., and the emulsion thickness was 30 um. A DP-320 screen printer (Newlong Seimitsu Kogyo Co., Ltd.) was used. The printed patterns were baked at 90 °C for 30 min in an oven (OFW-600V, AZ-1 Co., Ltd.). (b) The fabrics were a knit (AlphaWipe TX1009, Texwipe) and organdie. The fabric circuit board consisted of stretchable fabric and a PU sheet with a silver paste pattern. The fabric and PU sheet were thermally bonded using 100-μm-thick thermoplastic polyurethane film (SHM101, Sheedom Co., Ltd.). A hot press machine (JL-CO005B, Quick Art Co., Ltd.) was used to press the samples at 120°C for 15 s at a pressure of approximately 20 kPa. (c) LEDs and sensor components were mounted on a double-sided flexible substrate (Unicraft, 100 um thickness polyimide film and 18 μm copper foil) using standard solder paste (M705-ULT369, Senju Metal Industry Co., Ltd.). The double-sided flexible substrate with electronic components was thermally bonded to a fabric circuit board using the thermoplastic polyurethane film interposer. Via holes were drilled in the HMS film by using a laser cutting machine (VLS4.60-30, Universal Systems). The double-sided flexible and fabric circuit boards were connected by injecting silver paste into the via holes. The conventional structure had a single-sided flexible substrate on which the LEDs and sensors were soldered. A single-sided flexible board was bonded to the fabric circuit with adhesive (AX-040, Cemedine Corporation). The pads of the single-sided flexible board and the fabric circuit board were connected with stretchable silver paste in the horizontal direction.

### Model of the mechanical properties of knit and polyurethane film and FEM simulation

The stress concentrations in the proposed and conventional mounting structures under more than 30% strain were analyzed using the finite element method (FEM) software (SIMULIA Academic Abuqus 2019 software, Dassault Systèmes). The finite element model treated the electronic components embedded in the FPC as rigid bodies and treated the elastic knit and polyurethane film as hyperelastic materials^25^. Thermoplastic polyurethane films (40 mm long, 10 mm wide, and 800 µm thick knit and 40 mm long, 10 mm wide, and 280 um thick) were tested with a tensile test machine (FTN1-13A, Aiko Engineering Co., Ltd.). A reduced polynomial model^26^ was used to describe the deformation behavior of the knit and thermoplastic polyurethane film, because it is a strain-energy function with ${I}_{1}$ as a variable, and the accuracy of the model does not decrease significantly when there is no test data of multiple deformation modes and the material properties are calculated with the following Eq. (1).

1 $$ \begin{array}{*{20}l}    {W = \sum\limits_{{i = 1}}^{N} {C_{i} \left( {I_{1}  - 3} \right)^{i} } } \hfill  \\   \end{array}  $$

where $N$ and ${C}_{i}$ are the order of the model and the material parameter, respectively. The material parameters of the first- to fourth-order reduced polynomial model were calculated from the test data of the knit and thermoplastic polyurethane film. The results of the curve fitting are shown in Fig. 1S. The material parameters of the reduced polynomial models for the knit and HMS are shown in the table at the bottom of the figure. The coefficient of determination *R*^2^ was calculated to evaluate the accuracy of the model against the test data. In consideration of the accuracy of the model and the speed of the simulation, the deformation behavior of the textile substrate could be expressed with a reduced polynomial model of the third order.

**Figure 2:**
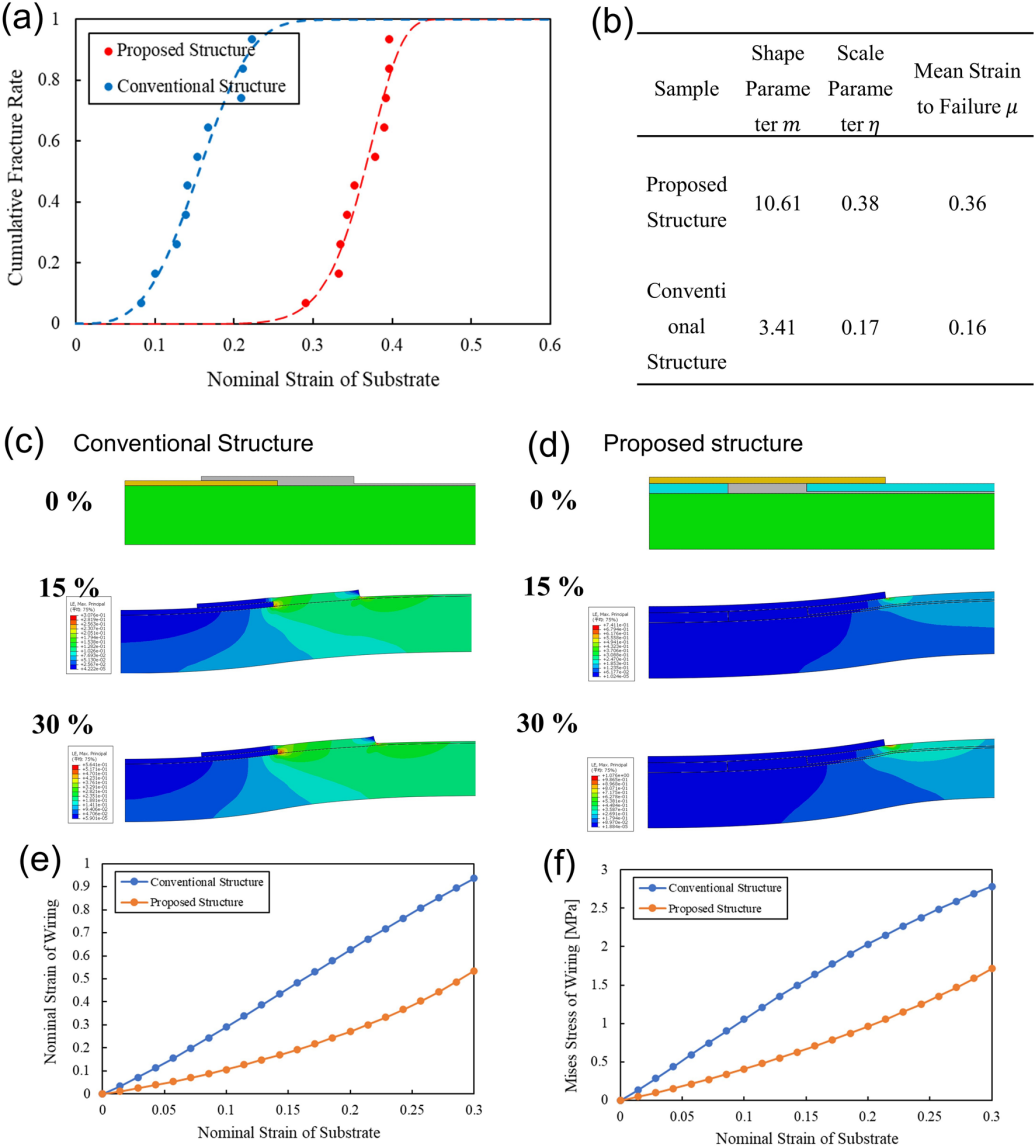
(**a**) Relationship between strain applied to electronic textile and cumulative failure. (**b**) Table of Weibull parameters of the conventional and proposed structure. (**c,e**) Distribution and plot of maximum principal strain of the conventional packaging structure. (**d,f**) Distribution and plot of maximum principal strain of the proposed packaging structure.

The simulation models of the electronic textile packaging with the conventional structure and the proposed structure are shown in Fig. 2S. A two-dimensional half model of the test piece cross section was used for symmetry. The conventional structure consisted of 10-mm-long and 700-μm-thick textile substrate, 8-mm-long and 25-μm-thick silver paste conductive paste, and 2-mm-wide and 60-μm-thick FPC. The proposed structure consisted of 10-mm-long and 100-μm-thick thermoplastic polyurethane film was inserted between the FPC and textile substrate.

The mesh size and shape of the boundary area between the FPC and thermoplastic polyurethane film was 10 μm and triangle elements, respectively. On the other hand, the mesh size and shape of the other areas were 50 μm and quadrilateral elements, respectively. In the boundary conditions, the horizontal and vertical displacements of the left side of the half model test piece were constrained. In addition, the right end of the half model test piece was given a displacement that was up to 30% of the length of the structure. Young's modulus and Poisson's ratio of the silver paste were 4 MPa and 0.49, respectively, while those of the FPC were 3000 MPa and 0.3, respectively. The mechanical stress–strain models of knit textile and thermoplastic polyurethane film were described in the Fig. 1S. The three-order reduced polynomial model parameters of the knit textile are ${C}_{1}=4.69 {e}^{2}$ MPa, ${C}_{2}=2.49 {e}^{2}$ MPa and ${C}_{3}=6.92 {e}^{1}$ while the three-order reduced polynomial model parameters of thermoplastic polyurethane film are ${C}_{1}=1.15 {e}^{3}$ MPa, ${C}_{2}=-1.67 {e}^{2}$ MPa and ${C}_{3}=1.60 {e}^{1}$. The convergence decision for this simulation was made using the Newton–Raphson method with incremental calculations and default convergence criterion.

### Weibull analysis of the failure strain

The Weibull distribution^27,28^ was applied to the rupture strain of the e-textile packaging structure. The cumulative Weibull distribution function $F\left(\varepsilon \right)$ is

2 $$\begin{array}{c}F\left(\varepsilon \right)=1-exp\left[-{\left(\frac{\varepsilon }{\eta }\right)}^{m}\right]\end{array}$$

where *ε,* m, and $\eta$ represent failure strain, shaper parameter, and < what else?? > . Taking the logarithm of Eq. (2) twice on both sides yields

3 $$\mathrm{lnln}\left(\frac{1}{1-F\left(\varepsilon \right)}\right) =m\mathrm{ln}\varepsilon -m\mathrm{ln}\eta $$

The Weibull parameters m and *η* were calculated from the slope a and the intercept b of the graph plotting the cumulative failure rate F(ε) calculated from the experimental results where the vertical axis is $\mathrm{lnln}\left(1/1-F\left(\varepsilon \right)\right)$ and the horizontal axis $\mathrm{ln}\varepsilon$. The cumulative failure rate F(ε) was calculated from the experimental results by using the median rank method.

4 $$\begin{array}{c}F\left(\varepsilon \right)=\frac{r-0.3}{n+0.4}\end{array}$$

where *r* is the cumulative number of failures and n is the number of test pieces. The average life

5 $$\begin{array}{c}\mu =\eta \Gamma \left(1+\frac{1}{m}\right)\end{array}$$

time μ was calculated from the calculated Weibull parameters by using the following equation.

## Results

### Experiments and simulations comparing conventional and proposed e-textile packaging structures

Experiments and numerical simulations were conducted to show that the proposed structure can withstand a larger expansion than the conventional structure can. The required mechanical strength of e-textiles is approximately 30%, which is equivalent to the stretchability of human skin. Therefore, the proposed structure was experimentally evaluated to withstand a stretching rate of more than 30%. In addition, the stress concentration on the packaging structure during expansion was analyzed in a nonlinear material mechanics simulation to verify that the stress concentration moved from the silver paste of the fabric circuit to the insulating thermoplastic polyurethane of our interposer, as proposed. For the experiments, e-textile packaging with the conventional and proposed structures were fabricated and tested using a tensile testing machine (FTN1-13A, Aiko Engineering Co., Ltd.). During the tensile test, the change in resistance of the fabric circuit was measured using a Keithley 2400 source meter. In the comparative experiments, the thickness *t* of the thermoplastic polyurethane film of the interposer was 100 μm, since this is the most commonly available thickness. The failure strain of the structure due to tension was assessed by performing a Weibull analysis. Specifically, the probability of fabric circuit failure was approximated by a Weibull distribution, which statistically describes the strength of materials, and the shape parameter, scale parameter, and mean strain in failure were obtained.

Figure 2a is a plot of the fracture strain on the horizontal axis and the cumulative failure rate of the proposed and conventional structures on the vertical axis. A fracture of silver paste wiring has a probability distribution of a Weibull distribution. Therefore, the cumulative fracture rate is the ratio of the number of fracture samples at a given strain to the number of all samples in the tensile test. One hundred samples for each of the conventional and proposed packaging structures were tested. From these distributions, we obtained the fracture strains based on Weibull analysis. The Weibull parameters, which indicate the characteristics of fracture, were obtained from the Weibull distribution equation described in the method section. The Weibull parameters calculated from the data are shown in the Fig. 2b. The geometry parameter *m* indicates the failure mode; *m *< 1 indicates an early failure, and *m *> 1indicates a wear-out failure. The conventional and proposed structures have *m* = 10.61 and 3.41, respectively; i.e., both fail in wear-out mode. Therefore, the fabric circuit with stretchable silver paste will break under tension and the electric resistance increases, which shows the packaging structural limit. The average lifetime of the proposed structure is 0.36 in strain, while that of the conventional structure is 0.16 in strain. Therefore, the proposed structure should be able to withstand up to 36% strain, which is more than the required 30% strain.

Figure 2c shows the distribution and plot of the maximum principal logarithmic strain of the conventional packaging structure and Fig. 2d shows the same for the proposed structure. In the experiment, the proposed structure has a larger fracture strain than the conventional structure. This is because the proposed structure reduces the strain to the wiring area compared to the conventional structure. Even when small strain of 15% is applied to the entire fabric, the strain is concentrated in the wiring area and the wiring fails. Since the fracture strain for the entire fabric can be obtained from experiments, but the wiring area cannot be measured, the stress and strain on the wiring area were evaluated using FEM simulations. Since the wiring material of elastic silver paste used in this study fractures at 50% strain, we investigated the strain of the entire fabric which induces more than 50% strain of the wiring area. In addition, the fracture property of elastic silver paste is represented by strain, not by stress in general, because elastic silver paste is a wiring material designed for high stretchability. Therefore, maximum principal logarithmic strains of proposed and conventional structures are shown in Fig. 2c,d. In the conventional structure, as the strain increases from 0%, 15%, and 30%, stress concentration is applied to the boundary between the electronic-component-embedded FPC and the fabric circuit with silver paste. When strain of 16% is applied, approximately 50% of that strain is concentrated in the silver paste wiring at the boundary, causing the fabric circuit wiring to rupture, as shown in Fig. 2e. In the proposed structure, the stress concentration is in the thermoplastic polyurethane film of the interposer where the silver paste wiring of the fabric circuit is not present. This is because the silver is located below the thermoplastic polyurethane interposer and the stress on the interconnects is reduced. When strain of 16% is applied to the entire fabric, the wiring does not rupture because only about 20% of the strain is applied to the wiring. On the other hand, when the entire fabric is subjected to strain of 30%, the wires fail, as shown in Fig. 2d.

Figure 2e,f shows the stress on the silver paste wiring of the fabric circuit for the proposed and conventional structures. At a strain of 16% on the substrate, the wiring of the conventional structure is subjected to a stress of 1.5 MPa, causing the wiring to rupture. In the proposed structure, only 0.6 MPa of stress is applied to the wiring at a substrate strain of 16%. Therefore, our packaging structure reduces the stress concentration on the silver paste wiring of the fabric circuit, which leads to our structure having higher stretchability.

### Optimization of stress-concentration-relocation packaging structure

**Figure 3:**
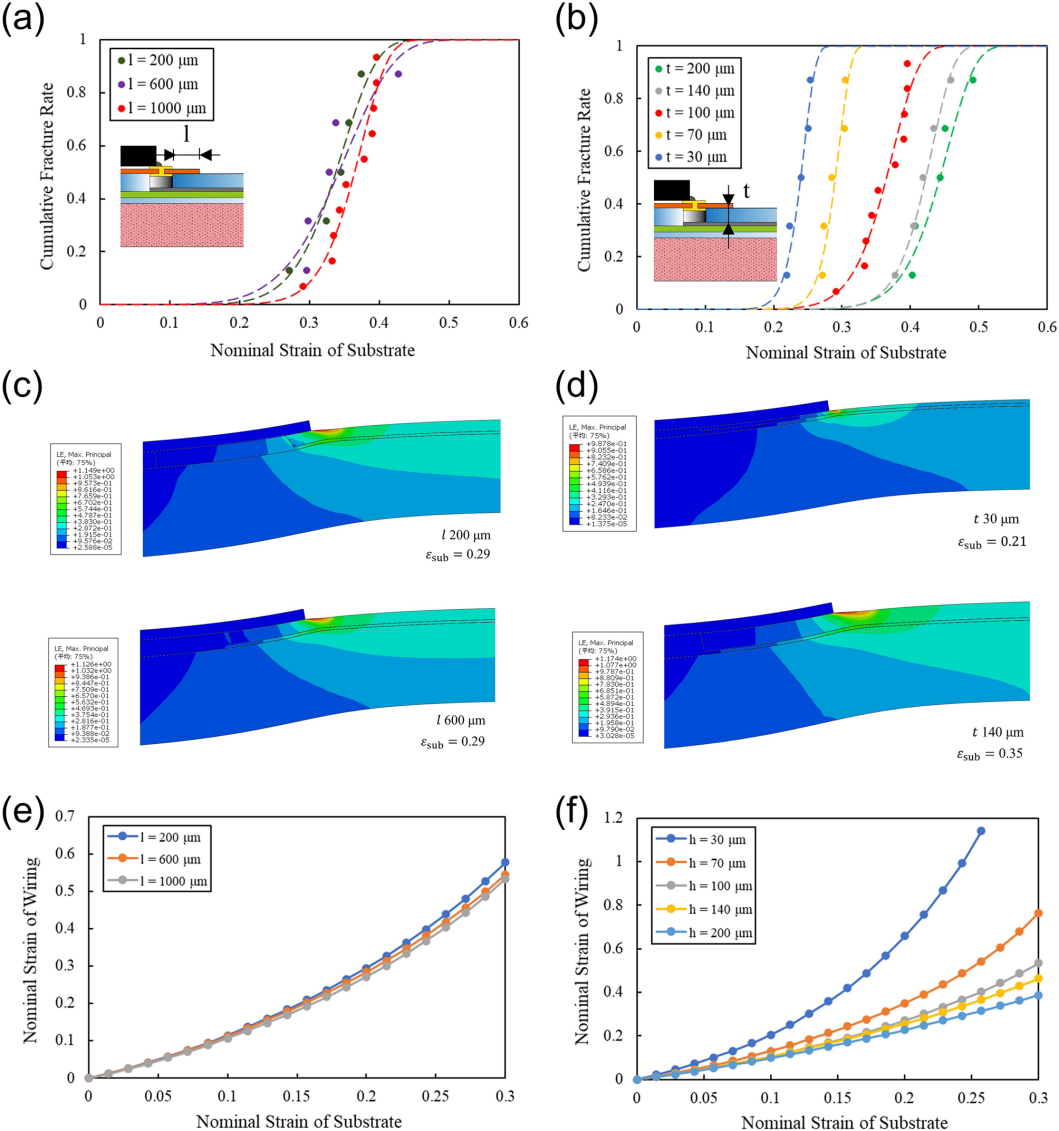
(**a,b**) Relationship between strain applied to the electronic textile and cumulative failure of proposed packaging structure for different distances* l* and different thermoplastic thicknesses *t.* (**c,e**) Distribution and plot of maximum principal strain of the proposed packaging structure for different distances* l* under applied strain. (**d,f**) Distribution and plot of the maximum principal strain of the proposed packaging structure for different thermoplastic thicknesses *t* under applied strain.

The relocation of the stress concentration from the silver paste wiring to the insulation layer of the thermoplastic polyurethane interposer is determined by the dimensions of the thermoplastic polyurethane interposer. The geometry of the interposer that can be optimized to relocate the stresses concentration are the thickness and diameter of the interposer, and the placement of the interposer in the packaging structure. The placement of the interposer is changed by the distance between the via holes and edge of the electronic-component-embedded FPC. Among these parameters, the diameter of the interposer is difficult to change because it is defined by the footprint of the electronic component. Therefore, test samples of the structure were fabricated with varying thicknesses *t* of thermoplastic polyurethane and distances *l* between the via holes and edge of the electronic-component-embedded FPC, as shown in Fig. 3a,b, and tensile strength tests were conducted. The stress concentration on the wiring of fabric circuit was also evaluated in an FEM simulation.

Figure 3a plots the rupture strain on the horizontal axis and the cumulative failure rates of the proposed and conventional structures on the vertical axis for different distances *l*. Because the accuracy with which the FPC can be cut is about 200 μm, *l* was varied between 200 and 1000 μm. The thickness of the film was 100 μm. The resultant shape parameter is m>1. Thus, the failure mode is wear and the lifetime indicates the structural limit. Varying *l* within the above range has no significant effect on the mean life and the strain at which the cumulative failure rate reached 10 %. Figure 3c,e shows the strain distribution and results of the FEM simulation with *l* as a parameter. The strain on the silver paste wiring of the fabric circuit relative to the strain applied to the entire fabric is almost the same even when *l* exceeds 200 μm. Therefore, the optimal distance *l* is more than 200 μm.

Figure 3b shows the probability density of fracture strain for different thicknesses *t* of the interposer. The thermoplastic polyurethane film of the interposer is made using a die-coating or balloon process in which thicknesses can range from 30 to 300 μm. Thus, the thicknesses *t* were varied within this range. In this case, the shape parameter is m > 1, which means the failure mode is wear and the lifetime indicates the structural limit. As *t* increases, the mean failure strain increases from 0.24 to 0.44. The increase in mean failure strain from *t* = 140 µm to *t* = 200 µm is less pronounced as the thickness *t* is varied. This is because the stress is reduced at locations far from the stress concentration. Figure 3d,f shows the strain distribution and a plot of results of the FEM simulation with the thickness *t* as a parameter. These figures show that the stress is reduced as *t* increases. In particular, the applied strain is significantly reduced when the thickness of the protective layer is 100 μm or more. Therefore, the proposed structure with thermoplastic polyurethane film more than 100-μm thick can reduce the stress more than the conventional structure can.

### Demonstration of mechanically reliable e-textile with integrated LEDs and MEMS sensors

**Figure 4:**
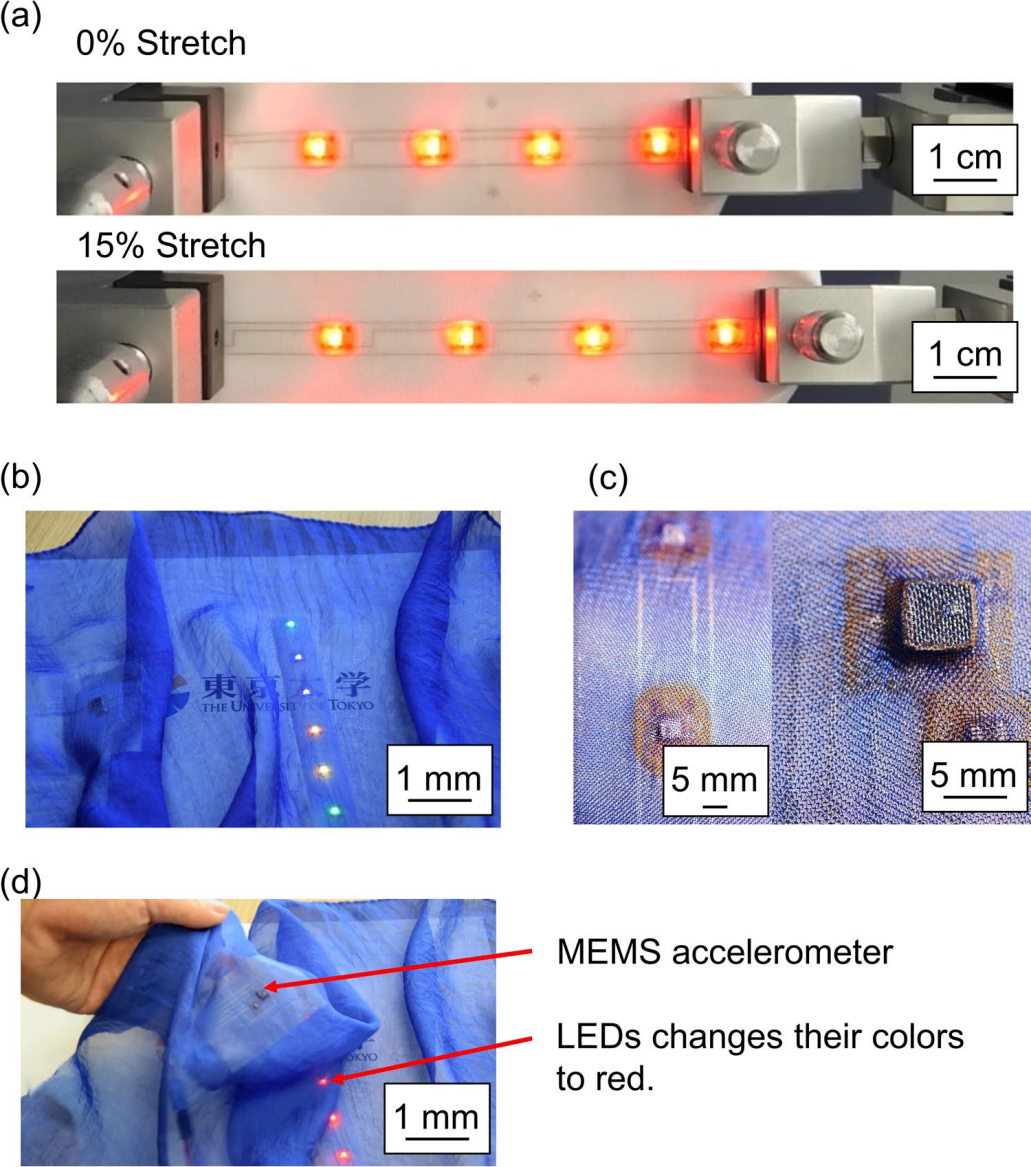
(**a**) Stretched LED-embedded e-textile with the proposed packaging structure. (**b**) e-textile integrating thin LEDs and MEMS sensors. (**c**) Magnified images of LED and MEMS sensors integrated into the fabric circuit structure. (**d**) The LEDs changed color to red by applying acceleration.

Figure 4 shows a prototype e-textile device incorporating LEDs (WS2812C-2020, Worldsemi Co., Limited) and accelerometers (ADXL316, Analog devices, Inc.) that was fabricated using our structure. Figure 4a shows that the LED fabric circuit with our e-textile packaging structure stretches 15%. Even if it stretches up to 30%, the e-textile packaging structure bears the applied strain. Figure 4b,c shows LED and MEMS accelerometers mounted on a very thin organdie fabric with a thickness of 300 μm. The LED changes its color to red depending on the applied acceleration (Fig. 4d). Thus, this structure for mounting electronic devices on fabric has excellent stretchability.

## Discussion

Our interposer in which thin thermoplastic polyurethane film with via holes can withstand a 36% expansion of e-textile devices simply by relocating stress concentration part from the wiring of the fabric circuit to the insulation film. Therefore, our structure can meet the requirement of the 30% elongation caused by conventional plain weaving textile structure. In addition, increasing the thickness of our interposer was effective in relocating the stress concentration part to the insulation film. However, the thickness of 100 μm is the limit of the present study, because conventional thermoplastic polyurethane film thickness is 100 μm and thicker film makes the fabric circuits stiff.

## Conclusions

We developed a thin stress-concentration-relocating interposer in which thin thermoplastic polyurethane film with via holes is inserted between electronic-component-embedded FPC and a fabric circuit with silver paste. Our interposer structure can withstand a 36% expansion of e-textile devices and thus meets the requirement of 30% stretchability for e-textile devices. An FEM simulation of the interposer structure indicated that the region in which stress concentrates when the fabric is stretched changes from the wiring of the fabric circuit in the conventional structure to the thermoplastic polyurethane film of the interposer; thereby the interposer provides higher stretchability compared with the conventional e-textile packaging. The optimal dimensions of the interposer require the thermoplastic polyurethane film to be more than 100 μm thick. The optimal interposer structure can be used to incorporate LEDs and MEMS sensors into e-textiles. It will lead to new mechanically reliable e-textile devices.

## Supplementary Information

Supplementary Figures.

## Data Availability

The datasets used and analyzed during the current study available from the corresponding author on reasonable request.

## References

[1] Ismar E, KursunBahadir S, Kalaoglu F, Koncar V (2020). Futuristic clothes: Electronic textiles and wearable technologies. Glob. Challenge.

[2] Xiong J, Chen J, Lee PS (2021). Functional fibers and fabrics for soft robotics, wearables, and human–robot interface. Adv. Mater..

[3] Wang D (2018). Chemical formation of soft metal electrodes for flexible and wearable electronics. Chem. Soc. Rev..

[4] Xue Z, Song H, Rogers JA, Zhang Y, Huang Y (2020). Mechanically-guided structural designs in stretchable inorganic electronics. Adv. Mater..

[5] Takamatsu S, Minami K, Itoh T (2021). Fabrication of highly stretchable strain sensor fiber by laser slitting of conductive-polymer-coated polyurethane film for human hand monitoring. Sens. Mater..

[6] Lim S (2015). Transparent and stretchable interactive human machine interface based on patterned graphene heterostructures. Adv. Funct. Mater..

[7] Rim YS, Bae SH, Chen H, De Marco N, Yang Y (2016). Recent progress in materials and devices toward printable and flexible sensors. Adv. Mater..

[8] Khan Y, Ostfeld AE, Lochner CM, Pierre A, Arias AC (2016). Monitoring of vital signs with flexible and wearable medical devices. Adv. Mater..

[9] Stoppa M, Chiolerio A (2014). Wearable electronics and smart textiles: A critical review. Sensors (Basel).

[10] Simegnaw AA, Malengier B, Rotich G, Tadesse MG, Van Langenhove L (2021). Review on the integration of microelectronics for e-textile. Materials (Basel).

[11] Yu X (2019). Skin-integrated wireless haptic interfaces for virtual and augmented reality. Nature.

[12] Ko Y (2019). Stretchable conductive adhesives with superior electrical stability as printable interconnects in washable textile electronics. ACS Appl. Mater. Interfaces.

[13] Araki T, Nogi M, Suganuma K, Kogure M, Kirihara O (2011). Printable and stretchable conductive wirings comprising silver flakes and elastomers. IEEE Electron. Dev. Lett..

[14] Chun KY (2010). Highly conductive, printable and stretchable composite films of carbon nanotubes and silver. Nat. Nanotechnol..

[15] Cho H (2019). Stretchable strain-tolerant soft printed circuit board: A systematic approach for the design rules of stretchable interconnects. J. Inf. Display.

[16] Di Vito D, Mosallaei M, Khorramdel B, Kanerva M, Mantysalo M (2020). Mechanically driven strategies to improve electromechanical behaviour of printed stretchable electronic systems. Sci. Rep..

[17] Liu Y, Pharr M, Salvatore GA (2017). Lab-on-skin: A review of flexible and stretchable electronics for wearable health monitoring. ACS Nano.

[18] de Mulatier S, Nasreldin M, Delattre R, Ramuz M, Djenizian T (2018). Electronic circuits integration in textiles for data processing in wearable technologies. Adv. Mat. Technol..

[19] Libanori R (2012). Stretchable heterogeneous composites with extreme mechanical gradients. Nat. Commun..

[20] de Mulatier S, Ramuz M, Coulon D, Blayac S, Delattre R (2019). Mechanical characterization of soft substrates for wearable and washable electronic systems. APL Mater..

[21] Matsuhisa N (2015). Printable elastic conductors with a high conductivity for electronic textile applications. Nat. Commun..

[22] Jahanshahi A (2013). Stretchable circuits with horseshoe shaped conductors embedded in elastic polymers. Jpn. J. Appl. Phys..

[23] Libanori R, Münch FHL, Montenegro DM, Studart AR (2012). Hierarchical reinforcement of polyurethane-based composites with inorganic micro- and nanoplatelets. Compos. Sci. Technol..

[24] Graz IM, Cotton DPJ, Robinson A, Lacour SP (2011). Silicone substrate with in situ strain relief for stretchable thin-film transistors. Appl. Phys. Lett..

[25] Ogden RW (1972). Large deformation isotropic elasticity—Correlation of theory and experiment for incompressible rubberlike solids. Proc. R. Soc. Lond. Ser. Math. Phys. Sci..

[26] Yeoh OH (1993). Some forms of the strain-energy function for rubber. Rubber Chem. Technol..

[27] Cran GW (1976). Graphical estimation methods for Weibull distributions. Microelectron. Reliab..

[28] Stone GC, Heeswijk RGV (1977). Parameter estimation for the Weibull distribution. IEEE Trans. Electr. Insulat..

