# Supplementary material for: Stress concentration-relocating interposer in electronic textile packaging using thermoplastic elastic polyurethane film with via holes for bearing textile stretch
Source: Sci Rep. 2022 Jun 3;12:9269. doi: 10.1038/s41598-022-13493-7 (PMC9166807; doi:10.1038/s41598-022-13493-7)
Supplement: Supplementary file 1 — Supplementary Figures. [file 41598_2022_13493_MOESM1_ESM.docx]

## Supporting information

Stress concentration-relocating interposer in electronic textile packaging using thermoplastic elastic polyurethane film with via holes for bearing textile stretch

**
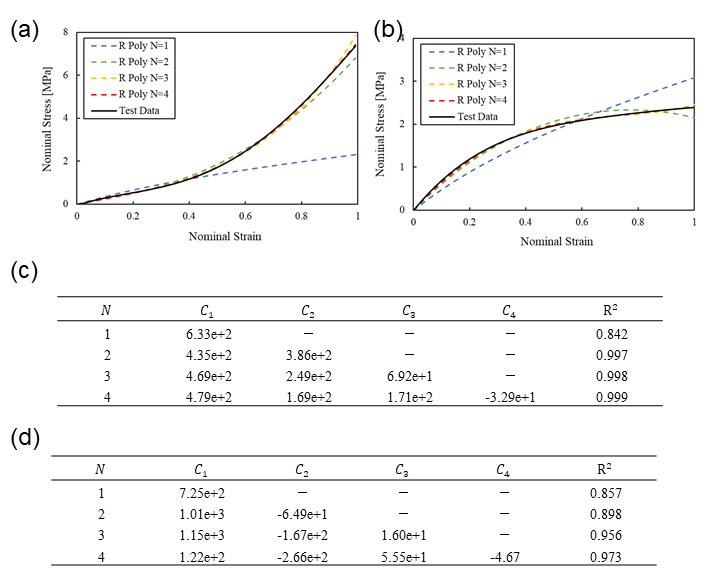
**

**Figure 1S.** (a) Experimental result of knit textile and curve fitted result of first- to fourth-order reduced polynomial model. (b) Experimental result of thermoplastic polyurehtane film and curve fitted result of first- to fourth-order reduced polynomial model. (c) Table of first- to fourth-order reduced polynomial model parameters and coefficient of determination of the knit textile. (d) Table of first- to fourth-order reduced polynomial model parameters and coefficient of determination of the thermoplastic polyurehtane film.


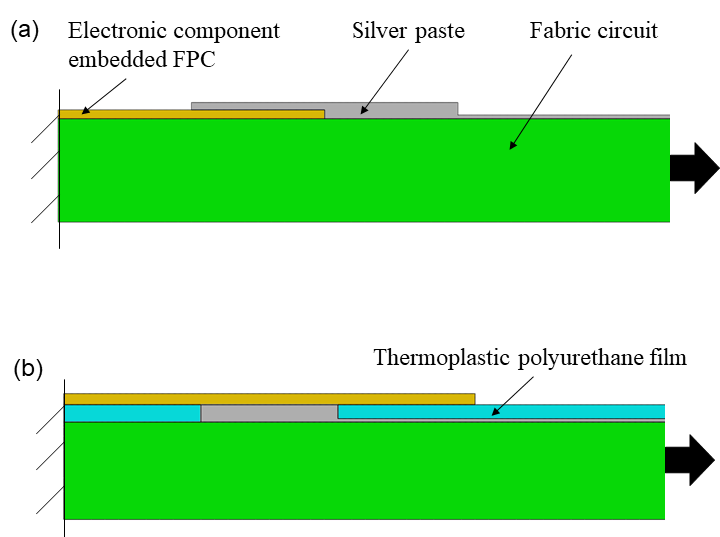


**Figure 2S.** (a) (b) Simulation models of electronic textile packagings with conventional structure and proposed structu
